# Exploring the Effect of Genetic Testing on Personalised Treatment Plans for Depression

**DOI:** 10.62641/aep.v54i1.2098

**Published:** 2026-02-15

**Authors:** Shichao Li, Liang Peng, Yuting Wang

**Affiliations:** ^1^Psychiatry Department, Suizhou Hospital Affiliated to Hubei University of Medicine, 441300 Suizhou, Hubei, China; ^2^Psychiatry Department, Suixian People's Hospital, 441315 Suizhou, Hubei, China

**Keywords:** genetic testing, depression, *CYP2C19*, sertraline

## Abstract

**Background:**

As a result of individual genetic variations, some patients show no response to initial antidepressant medications. This study aims to investigate the association between specific genetic polymorphisms and the efficacy of antidepressant drugs and to improve the accuracy and effectiveness of treatment under the guidance of genetic testing.

**Methods:**

A retrospective screening was conducted on medical records from, Suixian People's Hospital between January 2022 and December 2024. A total 202 patients with depression carrying the *CYP2C19* gene were selected after the application of exclusion criteria. They were assigned to three groups in accordance with their genetic metabolism types: the rapid metabolism group (Group A, n = 65), the intermediate metabolism group (Group B, n = 94) and the poor metabolism group (Group C, n = 43). All three groups were treated with sertraline for a six-week treatment cycle. The observation indicators included scores on the Hamilton Depression Scale (HAMD); onset time of drug effect; rates of response and remission; scores on the Clinical Global Impression–Improvement (CGI–I) scale; levels of the neurotransmitter factors 5-hydroxytryptamine (5-HT), γ-aminobutyric acid (GABA) and brain-derived neurotrophic factor (BDNF); incidence of adverse events; and scores on the Morisky Medication Adherence Scale-8 (MMAS-8).

**Results:**

The baseline data of the three groups of patients were comparable before medication (*p* > 0.05). Compared with those in Groups A and B, patients in Group C showed a significantly greater reduction in HAMD scores (all *p* < 0.05), along with higher response rates (all *p* < 0.05) and remission rates (all *p* < 0.05). Amongst the three groups, Group C had a shorter onset time of drug effect (all *p* < 0.05); more significant improvement in CGI–I scores (all *p* < 0.05); and more prominent upregulation of neurotransmitter factors, namely, 5-HT (all *p* < 0.05), GABA (all *p* < 0.05) and BDNF (all *p* < 0.05). Regarding the incidence of adverse events, Group C had the highest rate, whereas Group A had the lowest (10.8% vs. 24.5% vs. 41.9%). Compared with other groups, Group B exhibited a more significant increase in MMAS-8 scores (all *p* < 0.05).

**Conclusions:**

Metabolic phenotype exerts substantial effects on the therapeutic outcome of sertraline in patients with depression carrying the *CYP2C19* gene. Amongst groups, Group C showed better therapeutic efficacy but an elevated incidence of adverse events and lower medication adherence; Group A had relatively poor efficacy; and Group B demonstrated superior adherence. In clinical practice, individualised treatment can be implemented on the basis of *CYP2C19* metabolic typing to improve therapeutic efficacy and reduce adverse events and medical burden.

## Introduction

As a highly prevalent mental disorder with far-reaching harm worldwide, depression has evolved into a public health concern that constitutes a severe threat to human mental well-being. The World Health Organisation has published data revealing that approximately 322 million people across the globe are living with depression, with its incidence rate increasing year by year and its age of onset becoming increasingly younger [1]. In China, the prevalence rate of depression has reached 3.4% [2], and this figure continues to increase as social competition intensifies and the pace of life accelerates. Of note are the considerable disparities in the incidence of depressive disorders amongst different groups. For example, the prevalence rate of depressive disorders amongst adolescents has reached 7.4% [3], with heavy academic pressure and social barriers being the main contributing factors. Moreover, amongst women, the incidence rate of postpartum depression, which is closely related to fluctuations in hormone levels and poor adaptation to role transitions, is approximately 13.1%–16.6% [4]. Furthermore, amongst the elderly population, the prevalence rate of depression exceeds 20% because of factors, such as chronic diseases and loneliness [5], and the rate of detecting depression in empty-nest elderly individuals is nearly twice that in non-empty-nest elderly individuals. In patients, depression not only causes core symptoms, such as low mood, diminished interest and slowed thinking, but is also frequently associated with physical symptoms, including sleep disorders, changes in appetite and physical discomfort. In severe cases, it can lead to extreme behaviours, such as self-harm and suicide, thus imposing a heavy burden on patients themselves, their families and society [6]. From the perspective of social function, the work ability of patients with depression is markedly reduced: Their absenteeism rate is higher than that of healthy people, and their work efficiency decreases. Many patients are forced to resign or adjust their job positions because of their condition. In family life, patients’ emotional problems are likely to trigger family conflicts, resulting in strained marital relationships and alienated parent–child relationships. Families of patients with depression have varying degrees of emotional communication barriers, and in single-parent families, patients with depression have a considerable negative influence on their children’s growth [7].

The current treatment of depression still faces many challenges. Existing treatment regimens mainly have pharmacotherapy as their core, supplemented by psychotherapy, physical therapy and other methods. However, in terms of drug selection, most rely on the empirical judgment of clinicians, lacking an accurate guiding basis. As a result of marked genetic variations amongst individuals, different patients often show considerable diversity in the efficacy and tolerance of the same antidepressant [8]. Therefore, exploring precise treatment strategies to improve the effectiveness and safety of depression treatment has emerged as a pressing issue that demands urgent resolution in the field of psychiatry. With the rapid development of molecular biology and genomics technologies, genetic testing technology has been increasingly widely used in clinical medicine, providing fresh perspectives and strategies for the personalised management of depression. By analysing a patient’s gene sequence, genetic testing can identify gene variations related to drug metabolism and efficacy, thereby predicting the patient’s response to specific drugs and providing a scientific basis for clinicians to formulate individualised treatment plans [9]. As an important drug-metabolising enzyme system in the human body, the CYP450 enzyme system plays a key role in the metabolism of antidepressants. *CYP2C19*, an important member of the CYP450 family, participates in the metabolism of various antidepressants. Its genetic polymorphism can markedly affect the metabolic rate and blood concentration of drugs in the body, thereby influencing the efficacy of drugs and the occurrence of adverse events [10]. In recent years, genome-wide association studies have pinpointed more than 200 gene loci related to depression susceptibility and drug response. The research on drug-metabolising genes, such as *CYP2C19* and *CYP2D6*, is mature, and the detection accuracy for these genes has reached 80% in clinical verification [11]. The 2025 expert consensus on pharmacogenomic testing in psychiatry [12] explicitly recommends *CYP2C19* genetic testing for patients using selective serotonin reuptake inhibitors (SSRIs) to optimise treatment regimens. Sertraline, a commonly used SSRI, is widely employed in the treatment of depression because of its definite efficacy and high safety and is particularly commonly used in adolescent and elderly patients with depression. However, clinical practice has discovered that notable disparities exist in the therapeutic effects of sertraline amongst different patients. Specifically, some patients experience remarkable efficacy, whereas others have poor efficacy or experience obvious adverse events. Studies have shown that the *CYP2C19* gene polymorphism may be an important reason for this difference. *CYP2C19* exhibits diverse allelic variations, which can be divided into different phenotypes, such as rapid, intermediate and poor metabolisers, in accordance with their metabolic function [13]. Patients with different metabolic phenotypes have different metabolic capacities for sertraline, leading to differences in the *in vivo* exposure of the drug that in turn affect its therapeutic effect and risk of adverse events [14].

This study conducted a grouped research on patients carrying the *CYP2C19* gene and systematically analysed the differences in various indicators amongst patients with diverse metabolic phenotypes after sertraline treatment. It aims to shed light on the role of *CYP2C19* polymorphisms in shaping sertraline’s treatment effects, thereby furnishing newfound strategies and methods for the personalised treatment of depression. The outcomes of this study are expected to offer positive value in improving therapeutic effects for patients with depression and reducing the social influence of this condition.

## Materials and Methods

### General Information

**Fig. 1.**
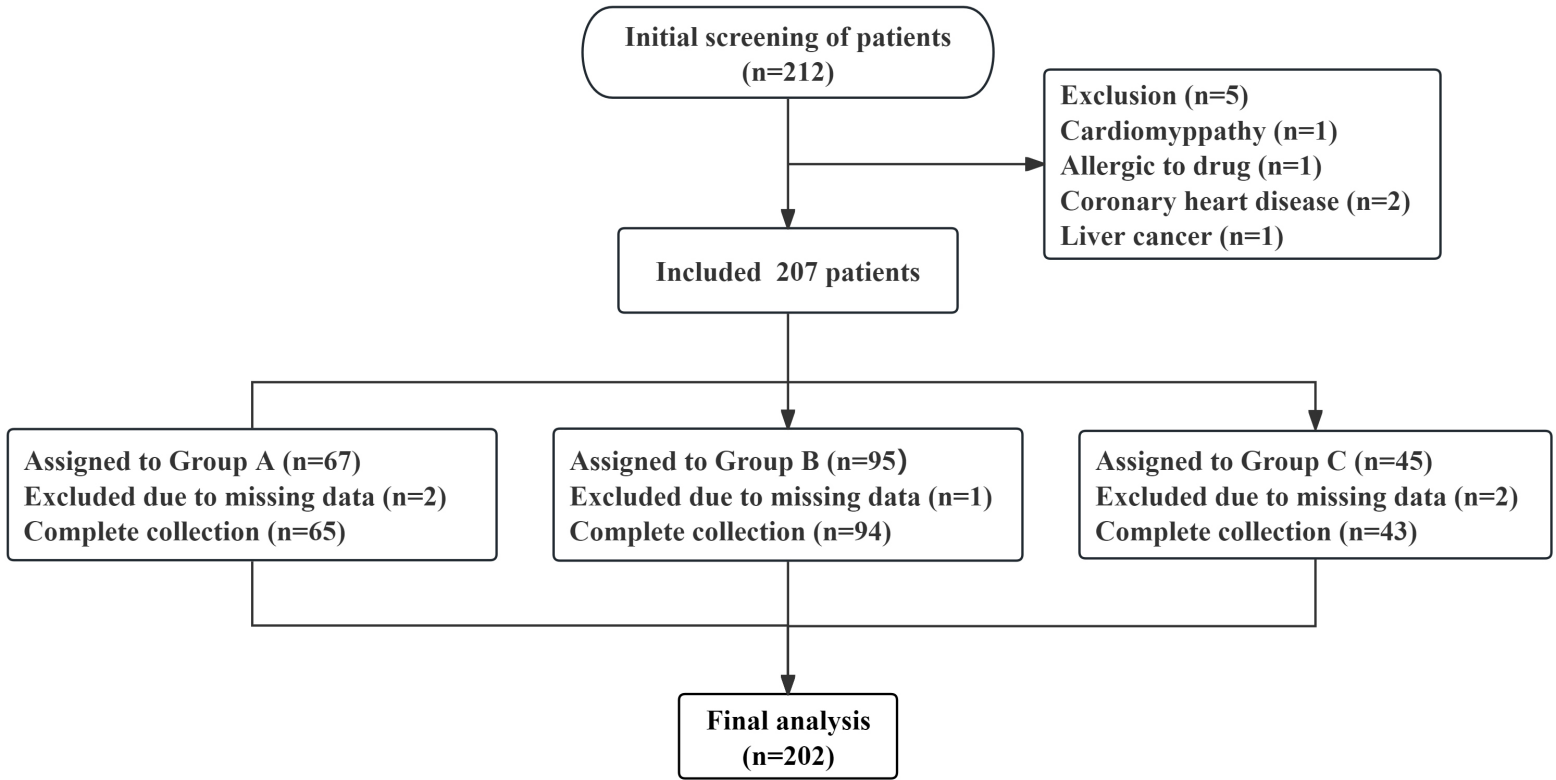
**Research flowchart**.

Medical records from Suixian People’s Hospital collected over the three-year period of January 2022–December 2024 were screened to select patients with depression who had previously sought treatment at this hospital. The primary objective of this study is to clarify the influence of *CYP2C19* polymorphisms on the therapeutic effect of sertraline. As shown in the study flow chart (Fig. 1), an initial screening identified 212 patients, and 207 patients were retained after exclusion criteria were applied. During the data analysis phase, five patients were excluded because of missing data in their medical records. Ultimately, 202 patients were included in the retrospective comparative analysis and divided into three groups on the basis of differences in alleles: the rapid metabolism group (Group A, n = 65), the intermediate metabolism group (Group B, n = 94) and the poor metabolism group (Group C, n = 43).

Overall, 212 patients were initially screened in this study. After the application of exclusion criteria, 207 patients were included, amongst which five had missing data. Ultimately, a total of 202 patients were analysed, with 65, 94 and 43 patients in Groups A, B and C, respectively.

### Inclusion Criteria

(1) Diagnosed with depression in accordance with the International Classification of Diseases, 11th Revision [15]. (2) Aged 18–60 years. (3) Medical records showing the completion of at least six weeks of sertraline treatment. (4) No use of antidepressants or antipsychotics within two weeks before admission, and receipt of antidepressant treatment after admission [16]. (5) With available *CYP2C19* genotype data.

### Exclusion Criteria

(1) Previous diagnosis of treatment-resistant depression. (2) Comorbidity with severe physical diseases. Patients are deemed to meet the exclusion criteria for severe physical diseases if they present with severe symptoms, such as severe pain (VAS ≥7 points), dyspnoea (manifested as a significant increase in respiratory rate, greater than 30 times per minute, accompanied with three obvious concave signs or cyanosis), massive bleeding (such as massive gastrointestinal bleeding, with blood loss exceeding 1000 mL in a short period of time, leading to hypotension and shock), or coma. (3) Current or past history of diseases that influence the absorption, distribution, or excretion of drugs (e.g., gastrectomy). (4) Organic diseases. (5) Pregnant women, lactating women and women who plan to become pregnant within two months. (6) Comorbidity with other mental illnesses. (7) History of allergy or abuse of the study drug. (8) Presence of suicidal ideation or behaviour. (9) Personal or family history of epilepsy. (10) Intellectual disability. (11) Participation in any clinical trial within the past two months [17].

### Treatment Methods

#### Genetic testing

Fasting peripheral venous blood was collected by using anticoagulant tubes, and the *CYP2C19* genotype was detected with a DNA microarray chip (SurePrint G3, Agilent Technologies Inc., Santa Clara, CA, USA). Patients were classified into three metabolic phenotype groups on the basis of the test results, as follows: Rapid metabolisers carry the *CYP2C19* *1/*17 or *17/*17 genotype; intermediate metabolisers exhibit the *CYP2C19* *1/*1, *1/*2, *1/*3, *2/*17, or *3/*17 genotype; and poor metabolisers present the *CYP2C19* *2/*2, *3/*3, or *2/*3 genotype. Ultimately, Group A (rapid metabolisers) included 65 patients, Group B (intermediate metabolisers) included 94 patients and Group C (poor metabolisers) included 43 patients [18].

#### Medication regimen

According to their medical records, all patients received sertraline hydrochloride (Zoloft, Pfizer Inc., New York, NY, USA) administered at an initial dosage of 25 mg/day. The dosage distribution ranges for Groups A, B and C were 75–150, 25–75 and 25–50 mg/day, respectively. The dosage of the drug used is based on the routine clinical treatment dosage and conforms to the dosage recommendation framework of the Chinese Guidelines for the Prevention and Treatment of Depressive Disorders (2024 edition) [2]. The observation period lasted for six weeks. During the entire clinical observation period, no other antidepressants, antipsychotics, psychotherapy, or physical therapy were administered concurrently.

### Outcome Measures

#### Hamilton Depression Scale Scores

The Hamilton Depression Scale (HAMD) is a commonly used tool for assessing depressive symptoms in clinical practice, with its 17-item version (HAMD-17) being one of the most widely applied [19]. HAMD-17 includes 17 symptom items, each categorised into different grades based on symptom severity, with most items scored on a scale of 0–4 or 0–2. A high total score indicates severe depression, with the following severity classifications: total score <7: no depressive symptoms; total score = 7–17: mild depression; total score = 18–24: moderate depression; and total score >24: severe depression.

#### Onset Time, Response Rate and Remission Rate

The time when the HAMD score first decreases by ≥20% compared with the baseline during treatment was used as the criterion for judging the onset time, suggesting that the drug may have produced an initial effect on patients [20]. Response is defined as a reduction of ≥50% in the HAMD score from baseline after six weeks of treatment, indicating that the patient’s depressive symptoms have improved significantly. The proportion of patients meeting this criterion amongst the total number of patients is the response rate. Remission is generally defined as HAMD score ≤7 after six weeks of treatment, indicating that the patient’s depressive symptoms have basically disappeared. The proportion of patients achieving this state amongst the total number of patients is the remission rate [21, 22].

#### Clinical Global Impression–Improvement

The Clinical Global Impression–Improvement (CGI–I) scale is a standardised assessment tool commonly used in psychiatric clinical research. It consists of two core dimensions: clinical global impression–severity (CGI–S) and CGI–I. Amongst these scales, CGI–I focuses on the magnitude of improvement in patients’ conditions after treatment relative to the baseline, serving as a key subjective indicator for evaluating therapeutic effects [23]. CGI–I adopts a seven-point rating scale, quantifying the range from ‘markedly worse’ to ‘markedly improved’; low scores indicate a good degree of improvement.

#### Neurotransmitters and Neurotrophic Factors

The neurotransmitters 5-hydroxytryptamine (5-HT) and γ-aminobutyric acid (GABA), as well as brain-derived neurotrophic factor (BDNF), were measured by using ELISA. Venous blood samples were collected from patients in the fasting state using anticoagulant tubes (2400423, Suzhou Lingyan Medical Technology Co., Ltd., Suzhou, Jiangsu, China). Serum supernatant was separated through centrifugation at 3000 rpm and room temperature for 10 min by using a centrifuge (Thermo Scientific, Sorvall ST 16R, Langenselbold, Hesse, Germany). Serum 5-HT concentration was measured with a ST/5-HT ELISA kit (D751013-0048, sensitivity: 9.38 ng/mL, Sangon Biotech, Shanghai, China). Serum BDNF concentration was quantified with a human BDNF ELISA kit (D711004-0048, sensitivity: 18.75 pg/mL, Sangon Biotech, Shanghai, China). Serum GABA content was measured with a GABA ELISA kit (ELK1513, sensitivity: 9.4 pg/mL, ELK Biotechnology, Wuhan, Hubei, China).

#### Incidence of Adverse Events

Adverse events linked to sertraline treatment for depression include gastrointestinal reactions, neurological reactions and sexual dysfunction [24]. The overall number of patients who had adverse events in each group was identified from medical records, and the incidence of adverse events for each group was calculated.

#### Morisky Medication Adherence Scale-8 Scores

The Morisky Medication Adherence Scale-8 (MMAS-8) is a commonly used tool for assessing patients’ medication adherence. It is particularly suitable for evaluating adherence in patients diagnosed with chronic medical conditions (e.g., hypertension, diabetes mellitus and depression). It consists of eight items, all answered with ‘yes’ or ‘no’, focusing on patients’ behavioural performance during medication treatment to reflect their adherence level comprehensively [25]. The scores of all items are summed, where the total score falls between 0 and 8, with high scores signifying good medication adherence.

### Sample Size Calculation Method

On the basis of a previous study [26], the effect size (Cohen’s *d*) was estimated to be 0.55. The statistical software G*Power (3.1.9.7, Heinrich–Heine–Universität Düsseldorf, Düsseldorf, North Rhine, Westphalia, Germany) was applied to determine the appropriate sample size. Calculation with the α value set at 0.05 and power at 80% and using a two-tailed test showed that 36 patients were required per group, thereby yielding a total sample size of 108 patients. In consideration of potential data loss in retrospective studies, the ultimate sample size for analysis in this study was 65 patients in Group A, 94 patients in Group B and 43 patients in Group C. These sample sizes met statistical requirements: the number of patients in each group is higher than the theoretically estimated 36 patients, and the total sample size (202 patients) is considerably larger than the theoretical total sample size (108 patients), ensuring the reliability and stability of the results.

### Statistical Methods

IBM SPSS Statistics (SPSS 27, IBM Corporation, Armonk, NY, USA) software was used for the statistical analysis of data. The Shapiro–Wilk test was applied for testing normality. Continuous data fitting a normal distribution were presented as mean ± SD. One-way ANOVA and ANCOVA test were employed for comparisons between groups, and paired *t*-test was used for comparisons within the same group before and after treatment. Effect size (η^2^) represents the magnitude of the difference between means between groups or between means before and after treatment within the same group. Bonferroni correction was used to adjust *p* values. Data that deviated from a normal distribution were expressed as M(IQR), and group-to-group comparisons were carried out via the Kruskal–Wallis test. Categorical data were presented as n (%), and the chi-squared test was used to compare groups. The 2 × 2 table, also known as the four-grid table, represents the effect size using the φ coefficient, whereas the n × m table represents the effect size using Cramer’s V to measure the correlation between categorical variables. Statistical significance for all data findings was determined as *p* < 0.05.

## Results

### Comparison of Patients’ Baseline Data

The baseline data of patients in this retrospective cohort were collected and analysed. These data included age, body mass index (BMI), gender, educational level, employment status, marital status, alcohol consumption history, smoking history, depression duration, current episode duration, first hospitalisation number, hospital stay length and depression severity. These data are presented in Table 1. A cross-group comparison of the data (amongst the three groups) demonstrated no statistically significant differences in the aforementioned baseline indicators (all *p* > 0.05), indicating that the three groups of patients were highly comparable and that comparisons of outcome measures could be conducted.

**Table 1.** **Baseline data (mean ± SD, n [%])**.

Variables	Group A	Group B	Group C	*p*	Effect size (η^2^/φ/Cramer’s *V*)	Total
	(n = 65)	(n = 94)	(n = 43)			
Age (years)	46.83 ± 7.93	46.34 ± 8.95	46.47 ± 8.15	0.936	0.001	46.52 ± 8.43
BMI (kg/m^2^)	24.04 ± 1.55	23.79 ± 1.68	24.28 ± 1.42	0.231	0.015	23.97 ± 1.59
Gender (male/female)	32 (49.2)/33 (50.8)	52 (55.3)/42 (44.7)	23 (53.5)/20 (46.5)	0.749	0.053	107 (53.0)/95 (47.0)
Education (elementary/high school/university)	10 (15.4)/39 (60.0)/16 (24.6)	20 (21.3)/49 (52.1)/25 (26.6)	6 (14.0)/25 (58.1)/12 (27.9)	0.773	0.067	36 (17.8)/113 (55.9)/53 (26.2)
Employment (unemployed/employed/retired)	20 (30.8)/25 (38.5)/20 (30.8)	37 (39.4)/32 (34.0)/25 (26.6)	10 (23.3)/24 (55.8)/9 (20.9)	0.138	0.131	67 (33.2)/81 (40.1)/54 (26.7)
Marital status (married/unmarried)	45 (69.2)/20 (30.8)	65 (69.1)/29 (30.9)	30 (69.8)/13 (30.2)	0.997	0.005	140 (69.3)/62 (30.7)
Alcohol	25 (38.5)	32 (34.0)	19 (44.2)	0.516	0.081	76 (37.6)
Smoking	26 (40.0)	36 (38.3)	15 (34.9)	0.865	0.038	77 (38.1)
Duration of depression (years)	4.85 ± 2.40	5.07 ± 2.35	4.93 ± 2.14	0.824	0.002	4.97 ± 2.32
Duration of current episode (months)	2.83 ± 1.36	2.80 ± 1.30	3.02 ± 1.41	0.649	0.004	2.86 ± 1.34
First time hospitalised	11 (16.9)	24 (25.5)	9 (20.9)	0.429	0.092	44 (21.8)
Duration of hospitalisation (days)	25.62 ± 5.11	25.76 ± 4.66	25.53 ± 5.63	0.968	<0.001	25.66 ± 5.00
Depression diagnosis (mild/moderate/severe)	15 (23.1)/31 (47.7)/19 (29.2)	21 (22.3)/54 (57.4)/19 (20.2)	7 (16.3)/25 (58.1)/11 (25.6)	0.594	0.083	43 (21.3)/110 (54.5)/49 (24.3)
HAMD scores	30.14 ± 2.83	29.99 ± 2.97	30.28 ± 2.75	0.854	0.002	30.10 ± 2.87

Note: BMI, body mass index; HAMD, Hamilton Depression Scale.

### Comparison of HAMD Scores Amongst Patients

The HAMD scores across the three groups were compared, with the results presented in Table 2. No significant difference in HAMD scores was found amongst the three groups before medication (*p* = 0.854). After medication, the HAMD scores of all three groups decreased significantly (all *p* < 0.05). Furthermore, postmedication comparisons revealed that the decrease in HAMD scores was more significant in Groups B and C than in Group A (Group B vs. Group A: *p* = 0.038; Group C vs. Group A: *p* < 0.001), and the decrease was more significant in Group C than in Group B (*p* = 0.032). The above results suggest that after medication, the depressive status of patients in all three groups improved. Amongst groups, Group C showed the best improvement, followed by Group B, whereas Group A showed the least improvement.

**Table 2.** **Comparison of HAMD scores (mean ± SD, scores)**.

Variables	Time	Group A	Group B	Group C	*p*	Effect size (η^2^/partial η^2^)
		(n = 65)	(n = 94)	(n = 43)		
HAMD scores	Before medication	30.14 ± 2.83	29.99 ± 2.97	30.28 ± 2.75	0.854	0.002
	After medication	12.55 ± 5.24^c^	10.73 ± 5.62^ac^	8.56 ± 5.47^abc^	0.041	0.025

Note: ^a^*p* < 0.05 vs. Group A; ^b^*p* < 0.05 vs. Group B; ^c^*p* < 0.05 vs. before medication. HAMD, Hamilton Depression Scale.

### Comparison of the Onset Time of Drug Effect

The onset time of sertraline after six weeks of treatment in the three groups is presented in Table 3. The onset time was 12.83 ± 3.36 days in Group A, 11.72 ± 3.32 days in Group B and 10.05 ± 3.52 days in Group C. A comparison of the onset time amongst the three groups revealed that Groups B and C had a shorter onset time than Group A (Group B vs. Group A: *p* = 0.025; *p* < 0.001), and Group C had a shorter onset time than Group B (*p* = 0.008). These results indicate that the onset time of sertraline varies amongst patients with different *CYP2C19* metabolic phenotypes: Group C had the fastest onset, Group A had the slowest and Group B had an onset time between those of the two other groups.

**Table 3.** **Comparison of onset time (mean ± SD, days)**.

Variables	Group A	Group B	Group C	*p*	Effect size (partial η^2^)
	(n = 65)	(n = 94)	(n = 43)		
Onset time	12.83 ± 3.36	11.72 ± 3.32^a^	10.05 ± 3.52^ab^	<0.001	0.021

Note: ^a^*p* < 0.05 vs. Group A; ^b^*p* < 0.05 vs. Group B.

### Comparison of Patients’ Response and Remission Rates

The drug response and remission rates of the three groups were summarised and compared, with the results presented in Table 4. The response and remission rates of Group A were 38.5% and 30.8%, respectively; those of Group B were 55.3% and 42.6%, respectively; and those of Group C were 74.4% and 62.8%, respectively. Group B exhibited higher response and remission rates than Group A (response rate: *p* = 0.037; remission rate: *p* = 0.034). Amongst the three groups, Group C had the highest response rate (Group C vs. Group A: *p* < 0.001; Group C vs. Group B: *p* = 0.033) and remission rate (Group C vs. Group A: *p* < 0.001; Group C vs. Group B: *p* = 0.028). The above results indicate that amongst the three groups of *CYP2C19* metabolic phenotypes under treatment with sertraline, Group C had the fastest response and remission, Group A had the slowest response and remission and the response and remission of Group B fell between those of the two groups.

**Table 4.** **Comparison of response rate and remission rate (n [%])**.

Variables	Group A	Group B	Group C	*p*	Effect size (Cramer’s *V*)
	(n = 65)	(n = 94)	(n = 43)		
Response rate	27 (38.5)	52 (55.3)^a^	32 (74.4)^ab^	0.001	0.259
Remission rate	20 (30.8)	40 (42.6)^a^	27 (62.8)^ab^	0.004	0.232

Note: ^a^*p* < 0.05 vs. Group A; ^b^*p* < 0.05 vs. Group B.

### Comparison of Patients’ CGI–I Scores

The scores for the global improvement component of the CGI scale are presented in Table 5. Before medication, no difference in CGI–I scores was found amongst the three groups (*p* = 0.831). After medication, a significant overall improvement was observed in each of the three groups (all *p* < 0.05). Amongst the three groups, Group C had the lowest CGI–I scores (Group C vs. Group A: *p* < 0.001; Group C vs. Group B: *p* = 0.021), and the CGI–I scores of Group B were only lower than those of Group A (*p* = 0.036). These results suggest that when comparing the effects of sertraline across different *CYP2C19* metabolic phenotypes, Group C exhibited the most significant improvement in therapeutic efficacy, whereas Group A showed poorer improvement compared with Groups B and C.

**Table 5.** **Comparison of CGI–I scores (mean ± SD, scores)**.

Variables	Time	Group A	Group B	Group C	*p*	Effect size (η^2^/partial η^2^)
		(n = 65)	(n = 94)	(n = 43)		
CGI–I score	Before medication	4.00 ± 1.85	3.94 ± 1.80	4.14 ± 1.81	0.831	0.002
	After medication	1.98 ± 0.82^c^	1.71 ± 0.82^ac^	1.42 ± 0.70^abc^	0.012	0.023

Note: ^a^*p* < 0.05 vs. Group A; ^b^*p* < 0.05 vs. Group B; ^c^*p* < 0.05 vs. before medication. CGI–I, Clinical Global Impression–Improvement.

### Comparison of Patients’ Neurotransmitter and Neurotrophic Factors

The statistical analysis of the data on the neurotransmitter factors 5-HT and GABA and the neurotrophic factor BDNF in the patients’ medical records is presented in Table 6. Before medication, the concentrations of 5-HT, GABA and BDNF in the three groups did not significantly differ (5-HT: *p* = 0.106; GABA: *p* = 0.197; BDNF: *p* = 0.677). After medication, the concentrations of these three indicators increased significantly in all three groups (all *p* < 0.05). Intergroup comparisons after medication revealed that compared with Group A, Groups B and C had significantly higher concentrations of 5-HT (Group B vs. Group A: *p* = 0.022; Group C vs. Group A: *p* < 0.001), GABA (Group B vs. Group A: *p* = 0.015; Group C vs. Group A: *p* < 0.001) and BDNF (Group B vs. Group A: *p* = 0.030; Group C vs. Group A: *p* < 0.001). Moreover, the concentrations of these three factors in Group C increased more significantly than those in Group B (5-HT: *p* = 0.026; GABA: *p* = 0.022; BDNF: *p* = 0.005). This result points to the ability of sertraline to improve neuronal dysfunction in patients, thereby alleviating depressive mood. Amongst the three *CYP2C19* metabolic phenotypes, the poor metabolic phenotype experienced the best sertraline therapeutic effect, whereas the rapid metabolic phenotype experienced worse sertraline therapeutic effects than the intermediate and poor metabolic phenotypes.

**Table 6.** **Comparison of 5-HT/GABA/BDNF (mean ± SD, ng/mL)**.

Variables	Time	Group A	Group B	Group C	*p*	Effect size (η^2^/partial η^2^)
		(n = 65)	(n = 94)	(n = 43)		
5-HT	Before medication	131.35 ± 0.23	131.41 ± 0.26	131.44 ± 0.31	0.106	0.019
	After medication	197.88 ± 1.56^c^	198.38 ± 1.58^ac^	198.95 ± 1.52^abc^	0.013	0.028
GABA	Before medication	121.35 ± 0.23	121.41 ± 0.26	121.39 ± 0.25	0.197	0.016
	After medication	182.88 ± 1.56^c^	183.38 ± 1.58^ac^	183.95 ± 1.52^abc^	0.022	0.038
BDNF	Before medication	15.97 ± 0.46	15.99 ± 0.43	15.92 ± 0.42	0.677	0.004
	After medication	25.98 ± 0.55^c^	26.19 ± 0.61^ac^	26.42 ± 0.57^abc^	<0.001	0.021

Note: ^a^*p* < 0.05 vs. Group A; ^b^*p* < 0.05 vs. Group B; ^c^*p* < 0.05 vs. before medication. 5-HT, 5-hydroxytryptamine; GABA, γ-aminobutyric acid; BDNF, brain-derived neurotrophic factor.

### Comparison of the Incidence of Adverse Drug Events

Table 7 shows the occurrence of adverse events in the three groups of patients. In Group A, three patients experienced gastrointestinal reactions, two had neurological reactions and two had sexual dysfunction. The total number of adverse events was seven, with an incidence rate of 10.8%. In Group B, 11 patients experienced gastrointestinal reactions, eight had neurological reactions and four had sexual dysfunction. The total number of adverse events was 23, with an incidence rate of 24.5%. In Group C, nine patients experienced gastrointestinal reactions, six had neurological reactions and three had sexual dysfunction. The total number of adverse events was 18, with an incidence rate of 41.9%. A comparison of adverse event occurrences amongst the three groups revealed that Group A had the lowest incidence of adverse events, whereas Group C had the highest (Group A vs. Group B: *p* = 0.030; Group A vs. Group C: *p* < 0.001; Group B vs. Group C: *p* = 0.039). Furthermore, the incidence of gastrointestinal adverse events was highest in Group B (*p* = 0.033), whereas no significant differences in neurological reactions or sexual dysfunction were found amongst the three groups (*p* = 0.188, *p* = 0.625). These results indicate that when patients with different *CYP2C19* metabolic phenotypes were treated with sertraline, those in Group A had the lowest incidence of adverse events, those in Group C had the highest incidence and the incidence rate of those in Group B was between the incidence rates of the patients in Groups A and B.

**Table 7.** **Comparison of adverse events (n [%])**.

Variables	Adverse events			
	Gastrointestinal reactions	Nervous system reactions	Sexual dysfunction	Total
Group A	3 (4.6)	2 (3.1)	2 (3.1)	7 (10.8)
(n = 65)				
Group B	11 (11.7)	8 (8.5)	4 (4.3)	23 (24.5)^a^
(n = 94)				
Group C	9 (20.9)	6 (14.0)	3 (7.0)	18 (41.9)^ab^
(n = 43)				
*p*	0.033	0.188	0.625	<0.001
Effect size (Cramer’s *V*)	0.184	0.146	0.068	0.262

Note: ^a^*p* < 0.05 vs. Group A; ^b^*p* < 0.05 vs. Group B.

### Comparison of Patients’ MMAS-8 Scores

Medication adherence during treatment was evaluated using MMAS-8 scores, with the results presented in Table 8. Before medication, no significant disparities in adherence scores were detected amongst the three groups (*p* = 0.469). After medication, the adherence scores in all three groups increased significantly (all *p* < 0.05), indicating that sertraline treatment alleviated patients’ depressive mood and improved their medication adherence. Furthermore, the increase in MMAS-8 scores was more significant in Group B than in Groups A and C (Group B vs. Group A: *p* = 0.011; Group B vs. Group C: *p* = 0.032), whereas no significant difference in MMAS-8 scores was found between Groups A and C (*p* = 0.929). The comparative results of the three groups indicate that sertraline improves patients’ depressive mood and enhances medication adherence. Amongst the groups, Group B showed the most significant improvement in adherence, whereas no significant difference was detected in the improvement in medication adherence between Groups A and C.

**Table 8.** **Comparison of MMAS-8 scores (mean ± SD, scores)**.

Variables	Time	Group A	Group B	Group C	*p*	Effect size (η^2^/partial η^2^)
		(n = 65)	(n = 94)	(n = 43)		
MMAS-8 scores	Before medication	3.31 ± 1.39	3.23 ± 1.39	2.98 ± 1.46	0.469	0.008
	After medication	6.00 ± 1.40^c^	6.55 ± 1.28^ac^	6.02 ± 1.34^bc^	0.025	0.022

Note: ^a^*p* < 0.05 vs. Group A; ^b^*p* < 0.05 vs. Group B; ^c^*p* < 0.05 vs. before medication. MMAS-8, Morisky Medication Adherence Scale-8.

## Discussion

The treatment of depression, which is a highly prevalent mental disorder worldwide, has long been a key focus and challenge in the field of psychiatry. Current treatment regimens largely rely on empirical medication. However, considerable genetic variations amongst individuals lead to nonresponse to initial medications in some patients. This situation not only delays the timing of treatment but also increases patients’ suffering and medical burden [27]. Against this backdrop, exploring precise treatment strategies has become critical to break through current bottlenecks in depression treatment. The CYP450 enzyme system, as the primary drug-metabolising enzyme system in the liver, is critical to the vivo transformation of drugs. Amongst its members, *CYP2C19*, a key component of the CYP450 family, has been confirmed to exhibit genetic polymorphisms that are closely associated with the metabolic efficiency of various drugs. The *CYP2C19* gene is located on chromosome 10q24.2; to date, multiple allelic variants have been identified [28]. Different genotypes exhibit remarkable differences in enzyme activity and can be used to classify the population further into rapid, intermediate and poor metabolisers. The in-depth investigation of the effect of *CYP2C19* metabolic phenotypes on the therapeutic effect of sertraline provides an important theoretical basis and practical reference for the personalised treatment of depression [29].

A large-scale study on the Chinese Han population conducted by Yan *et al*. [29] found that the serum concentrations of sertraline in those with poor and intermediate *CYP2C19* metabolism had increased relative to that in the group with normal *CYP2C19* metabolism. Additionally, the antidepressant conversion rates of sertraline in the poor and intermediate *CYP2C19* metabolism groups had increased significantly compared with that in the normal *CYP2C19* metabolism group. The results of the aforementioned study are in line with those of the present study. In the present work, compared with Groups A and B, Group C showed a significantly greater reduction in HAMD scores, along with higher response and remission rates, a shorter time to the onset of drug effects and more significant improvements in CGI–I scores. These results are associated with different alleles of *CYP2C19*. Patients with the rapid metabolic phenotype carry wild-type alleles of the *CYP2C19* gene, resulting in high enzyme activity and rapid drug metabolism and decomposition. When these patients take sertraline, the drug is rapidly metabolised in the body, making maintaining its blood concentration at an effective therapeutic level difficult; this situation may thus affect its therapeutic effect [30]. By contrast, patients with the poor metabolic phenotype mostly carry loss-of-function alleles of the *CYP2C19* gene, leading to a remarkable decrease in enzyme activity. As a result, the drug is metabolised slowly in the body, and the blood drug concentration easily increases and remains at a high level. *CYP2C19* enzyme activity in patients with the intermediate metabolic phenotype is between that in the rapid and poor metabolism groups, leading to a moderate rate of drug metabolism [10].

5-HT is a crucial central neurotransmitter that plays a key role in regulating physiological processes, such as mood, sleep and appetite. A large body of evidence [31] indicates that patients with depression exhibit reduced 5-HT function and a decline in 5-HT concentration within the synaptic cleft. This phenomenon is recognised as a critical mechanism underpinning the pathogenesis of depression. As an SSRI, sertraline exerts its therapeutic effect primarily by inhibiting the reuptake of 5-HT by neurons, thereby augmenting the concentration of 5-HT in the synaptic cleft and alleviating depressive symptoms [31]. The outcomes of the present study indicate that compared with those in the other groups, patients in Group C exhibited a more significant upregulation of 5-HT after sertraline treatment. This upregulation was strongly correlated with the high serum concentrations of sertraline in these patients. As a result of the low activity of the *CYP2C19* enzyme, sertraline is metabolised slowly in the body, allowing it to exert a sustained inhibitory effect on 5-HT reuptake. This effect causes a distinct increase in 5-HT levels within the synaptic cleft, thereby alleviating depressive symptoms effectively. By contrast, patients in Group A demonstrated rapid sertraline metabolism and low serum drug concentrations, indicating a weak inhibitory effect on 5-HT reuptake. Consequently, the degree of 5-HT upregulation was relatively low, which is an important reason for the poor therapeutic effect in this group. GABA is the primary inhibitory neurotransmitter in the central nervous system. It has various effects, such as sedation, anxiolysis and anticonvulsion effects. Studies have demonstrated that patients with depression present with diminished brain GABA levels and impaired GABA-mediated neurotransmission, which may be closely associated with the development of depressive symptoms [32]. In addition to its effects on the 5-HT system, sertraline may regulate the function of GABA neurons by increasing GABA release or reducing its metabolism to elevate GABA concentration in the synaptic cleft, thereby exerting an antidepressant effect [33]. In the present study, compared with other groups, Group C showed a more significant upregulation of GABA that was related to its higher serum concentration of sertraline. High concentrations of sertraline may effectively activate GABA neurons, promote GABA synthesis and release and simultaneously inhibit GABA degradation. This effect leads to a marked increase in GABA concentration in the synaptic cleft that enhances inhibitory neurotransmission, relieves patients’ anxiety and tension and improves depressive symptoms. Patients in Group A had a low degree of GABA upregulation because of the low serum concentration of sertraline and its weak regulatory effect on the GABA system. BDNF is recognised as a crucial neurotrophic factor that plays a critical role in the growth, differentiation, survival and repair of neurons and is essential for maintaining the normal function and plasticity of the nervous system [34]. It can promote neuronal regeneration and synaptogenesis and improve neural plasticity, thereby helping alleviate depressive symptoms and prevent disease recurrence [35]. The outcomes of the present study showed that patients in Group C experienced a more significant upregulation of BDNF than those in the other groups because the high serum concentration of sertraline in Group C can effectively stimulate the synthesis and release of BDNF. Sertraline may activate relevant signalling pathways (e.g., the mitogen-activated protein kinase pathway) to promote BDNF gene expression and increase BDNF levels [34]. The significant increase in BDNF levels can promote neuronal regeneration and synaptic remodelling in brain regions, such as the hippocampus, and improve neural plasticity and thereby enhance the antidepressant effect, leading to the improved clinical outcomes in patients in Group C [36].

Adverse drug events are important factors affecting patients’ treatment adherence and therapeutic efficacy [37]. In the present study, amongst groups, Group C had the highest incidence of adverse drug events. This finding was closely associated with the high serum concentration of sertraline in patients with the poor metabolic phenotype. Elevated serum drug concentrations may lead to drug accumulation in the body, thereby elevating the risk of adverse drug events, such as nausea, vomiting, dizziness and insomnia [38]. These adverse drug events not only cause physical discomfort in patients but may also reduce their confidence in treatment, resulting in decreased treatment adherence. By contrast, amongst groups, Group A had the lowest incidence of adverse drug events because patients with the rapid metabolic phenotype exhibit rapid drug metabolism and low serum drug concentrations, thereby mitigating the occurrence of adverse drug events [29]. Additionally, compared with other groups, Group B showed a more significant increase in MMAS-8 scores, indicating improved treatment adherence in patients with the intermediate metabolic phenotype. This result was obtained because during treatment, patients in Group B maintained serum drug concentrations within a relatively stable therapeutic range, whereas their incidence of adverse drug events was low [39]. This situation allowed patients to tolerate treatment well and adhere to medication regimens, thus maintaining good treatment adherence [40]. Patients with depression in the poor metaboliser group presented a clinical contradiction: although they achieved the best therapeutic efficacy, they had the highest incidence of adverse reactions and the worst medication adherence amongst patients. The core mechanism of this effect lies in the fact that their genotype causes the loss or remarkable reduction of sertraline-metabolising enzyme activity, leading to increased *in vivo* drug exposure and prolonged elimination half-life. Although high drug concentrations can fully inhibit 5-HT reuptake and upregulate GABA and BDNF levels, thereby delivering superior efficacy, the dose-dependent nature of sertraline’s adverse reactions indicates that high drug accumulation directly increases the risk of adverse events, such as gastrointestinal reactions and dizziness. Sustained experiences of adverse reactions then reduce patients’ confidence in treatment, triggering behaviours, like voluntary dose reduction and missed doses, that ultimately result in decreased medication adherence. Clinicians can balance efficacy and safety through genotype-guided individualised dose adjustments, hierarchical intervention for adverse reactions and full-course medication management to address the above contradiction.

Given that this study did not directly measure the concentration of sertraline in patients’ plasma, its current explanation for the mechanism underlying the aforementioned differences in therapeutic efficacy still relies on indirect inferences based on known theories regarding *CYP2C19* gene function and pharmacokinetics and lacks support from direct plasma drug concentration data. This result is believed to be associated with the distinct allelic characteristics of the *CYP2C19* gene [19]. This inference is primarily based on the fact that extensive metabolisers carry the wild-type alleles of the *CYP2C19* gene, which encode metabolic enzymes with high activity capable of rapidly catalysing the biotransformation of sertraline. In accordance with the basic principles of pharmacokinetics, when such patients take the standard dose of sertraline, the drug is rapidly metabolised and eliminated in the body. The concentration of the drug in plasma may be difficult to maintain within a continuously effective therapeutic window. This effect is likely a key reason for poor therapeutic efficacy. By contrast, poor metabolisers mostly carry loss-of-function alleles of the *CYP2C19* gene [13]. Mutations in these alleles lead to a remarkable reduction or even complete inactivation of metabolic enzyme activity [16]. In accordance with the theoretical logic of drug metabolism, the decrease in enzyme activity directly decelerates the metabolic rate of sertraline in the body. The drug can therefore be inferred to reach a high plasma concentration and maintained for a long duration. This relatively stable state of high drug concentration in plasma is likely the core mechanism underlying the good therapeutic efficacy and fast onset of action observed in the poor metaboliser group. Intermediate metabolisers typically have heterozygous genotypes of the *CYP2C19* gene, combining wild-type and loss-of-function alleles [30]. Their metabolic enzyme activity is exactly between those of extensive and poor metabolisers; consequently, their drug metabolism rate and inferred plasma drug concentration level also fall between those of the two groups [17]. This intermediate state logically corresponds to the moderate level of efficacy indicators observed in this patient group. Future studies should further incorporate indicators for the detection of plasma drug concentration to form a complete data chain. Such an approach will provide direct and conclusive mechanistic evidence for the differences in therapeutic efficacy amongst patients with differing metabolic phenotypes while also offering a precise basis for formulating individualised sertraline dosing regimens on the basis of metabolic phenotypes.

This study has the following limitations despite its findings: Covariance analysis notwithstanding, confounding factors remained. Its retrospective design, small single-hospital sample and lack of *CYP2C19* *1/*1 grouping compromise the reliability and generalisability of its results and accuracy of metabolic phenotype description. Additionally, it only examined *CYP2C19*–sertraline associations with a six-week period, which is insufficient for long-term efficacy assessment. Future research should use large multicentre prospective samples, explore multigene–antidepressant interactions to build prediction models, extend observation periods for long-term outcomes and integrate clinical/environmental factors for personalised plans.

## Conclusions

The *CYP2C19* metabolic phenotype has a significant effect on the therapeutic efficacy of sertraline in patients with depression carrying this gene. Great importance should be attached to the application of genetic testing in the personalised treatment of depression, and a reasonable treatment plan should be formulated on the basis of a patient’s *CYP2C19* metabolic genotype to improve therapeutic efficacy and reduce adverse events and medical burden. Meanwhile, further in-depth studies are needed in the future to improve personalised treatment strategies continuously and provide improved and efficient medical services for patients with depression.

## Availability of Data and Materials

The data supporting the findings of this study can be obtained from the corresponding author, upon request.
